# Understanding Performance Decrements in a Letter-Canceling Task: Overcoming Habits or Inhibition of Reading

**DOI:** 10.3389/fpsyg.2018.00711

**Published:** 2018-05-15

**Authors:** Larry Myers, Steven Downie, Grant Taylor, Jessica Marrington, Gerald Tehan, Michael J. Ireland

**Affiliations:** School of Psychology and Counselling, Institute for Resilient Regions, University of Southern Queensland, Ipswich, QLD, Australia

**Keywords:** ego-depletion, strength model, self-regulation, sequential task, letter-crossing

## Abstract

The importance of self-regulation in human behavior is readily apparent and diverse theoretical accounts for explaining self-regulation failures have been proposed. Typically, these accounts are based on a sequential task methodology where an initial task is presented to deplete self-regulatory resources, and carryover effects are then examined on a second outcome task. In the aftermath of high profile replication failures using a popular letter-crossing task as a means of depleting self-regulatory resources and subsequent criticisms of that task, current research into self-control is currently at an impasse. This is largely due to the lack of empirical research that tests explicit assumptions regarding the initial task. One such untested assumption is that for resource depletion to occur, the initial task must first establish an habitual response and then this habitual response must be inhibited, with behavioral inhibition being the causal factor in inducing depletion. This study reports on four experiments exploring performance on a letter-canceling task, where the rules for target identification remained constant but the method of responding differed (Experiment 1) and the coherence of the text was manipulated (Experiments 1–4). Experiment 1 established that habit forming and behavioral inhibition did not produce any performance decrement when the targets were embedded in random letter strings. Experiments 2–4 established that target detection was sensitive to language characteristics and the coherence of the background text, suggesting that participants’ automatic reading processes is a key driver of performance in the letter-e task.

## Introduction

Self-regulation is typically taken to refer to people’s ability to overcome their immediate impulses in order to achieve different outcomes and it is argued to be the cornerstone of all functional behavior (Duckworth and Seligman, 2005; Baumeister and Vohs, 2007; Moffitt et al., 2011). The capacity for self-control is thought to emerge from one’s temperament and social surroundings (Wills and Dishion, 2004); with proficiency—or deficiency—in one’s capacity for self-regulation having an impact at individual, interpersonal, and social levels (Mead et al., 2010). Those who are adept at self-regulating tend to exercise more, be academically successful, and have more satisfying interpersonal relationships (Kelly and Conley, 1987; Duckworth and Seligman, 2005; Moffitt et al., 2011). While those who show a relative inability to self-regulate make impoverished monetary, health and dietary decisions, as well as show an increased propensity for risky and criminal behaviors (Pratt and Cullen, 2000; Baumeister, 2002; Quinn and Fromme, 2010). Additionally, low levels of self-regulation can also predict increased symptoms of some psychological conditions such as post-traumatic stress disorder (Walter et al., 2010).

With much of people’s lives affected by self-regulatory ability, there has been great interest in explaining the mechanisms and antecedents of self-regulation and self-regulatory failure. Baumeister et al. (1998) proposed one influential theory known as the *limited-strength* model of self-regulation. In this model, people’s ability to self-regulate is governed by a limited pool of resources; such that repeated acts of self-regulation deplete this resource pool leaving the participant vulnerable to subsequent self-regulatory failure until this resource pool is replenished. This decline of self-regulatory efficacy with increased self-regulatory actions is known as the *ego-depletion* effect. An often-used metaphor is to liken self-regulation to a muscle, in that the use of a muscle leaves it fatigued and temporally reduces its effectiveness until sufficient time passes in which it can fully recuperate (Baumeister et al., 1998; Muraven et al., 1998).

Empirical evidence for the limited-strength model originated in two seminal articles (Baumeister et al., 1998; Muraven et al., 1998), which, across eight experiments, demonstrated that one’s ability to self-regulate was impoverished in cases following an activity that also required self-regulation. Aside from providing support for the limited-strength model, these studies also established the *sequential-task* paradigm as the standard means of exploring resource depletion in general, and the strength model in particular.

The sequential-task paradigm refers to the succession of experimental manipulations presented to research participants. In most cases, participants engage in two sequential tasks: an intervention task and an outcome task. The dependent variable is assessed using performance on the outcome task. The outcome task is identical across the treatment and control conditions and always involves some form of self-regulation. The intervention task varies between conditions. For the control condition, the intervention task does not require self-regulation. In the experimental or intervention condition, the intervention task does require some use of self-regulation. In the experimental condition, the intervention task and outcome task can vary along cognitive or emotional domains as long as both involve self-regulation. The guiding assumption of this approach is that all forms of self-regulation rely on a domain-general pool of resources. The paradigmatic example of this occurs within Baumeister et al.’s (1998) seminal article in which the participants in the experimental condition had to resist the urge to eat chocolate chip cookies and subsequently attempt to solve unsolvable geometric line puzzles. The authors found that those who had to resist the urge to eat the cookies spent less time and made fewer attempts to solve the puzzles before abandoning the task. This led Baumeister et al. (1998) to conclude that self-regulating eating exhausted the general resource pool that were needed for persistence on the cognitive outcome task.

### Conflicting Evidence for Ego-Depletion

Hagger et al. (2010) provided strong empirical support for the limited-strength model through a meta-analysis of 83 studies (comprising 198 individual tests) from which they concluded that the ego-depletion effect was reliable and represented a medium to large effect size, though they did note the effect was heterogeneous. However, when Carter and McCullough (2014) reanalyzed the Hagger et al. (2010) data they found that if small study effects and publication bias were accounted for then the “results do not support the claim that the depletion effect is meaningfully different from zero” (Carter and McCullough, 2014, p. 7). Furthermore, Carter et al. (2015) conducted another meta-analysis using stricter inclusion criteria (e.g., not including extension studies or those that used uncommon and ‘questionable’ intervention tasks), incorporating unpublished studies, and tested if subsequent effects are domain-general as the strength model assumes or restricted to specific cognitive domains. A reliable ego-depletion effect did emerge, however, it was restricted to one specific type of outcome task, that of standardized test scores. With all other outcome tasks their analysis suggested that the average ego-depletion effect was “indistinguishable from zero” (Carter et al., 2015, p. 16).

In addition to questions raised by these meta-analyses, researchers have reported difficulty in replicating findings using pre-registered studies and with diverse, large samples. Both Xu et al. (2014) and Lurquin et al. (2016) reported non-significant results despite using the most reliable experimental procedures and having large sample sizes to detect the effect. These critiques and replication failures of ego-depletion spurred a pre-registered, multi-lab replication study published by Hagger and Chatzisarantis (2016). The study used 23 independent labs and 2,141 participants to replicate a study by Sripada et al. (2014), which previously found a significant depletion effect (*d* = 0.69). The study employed a letter-crossing task as the intervention task and the Multi-Source Interference Task as the outcome task. The data from this trial failed to show a depletion effect significantly different from zero. Hagger and Chatzisarantis (2016) comment that this finding concurs more with Carter et al.’s (2015) critical meta-analytic estimate than Hagger et al.’s (2010) favorable meta-analytic estimate.

These findings are, however, not conclusive. Carter et al. (2015) note that while their analysis did include a large amount of unpublished data, there was still more data unavailable for analysis, thereby making their statistical corrections only speculative. Moreover, it has been argued that the bias-corrected estimates used in Carter et al.’s (2015) critical meta-analysis may not be a reliable correction for publication bias, especially if the effect is heterogeneous (Inzlicht et al., 2015; Reed et al., 2015). Additionally, Baumeister and Vohs (2016) criticized the multi-lab replication study on methodological grounds. They argued that the letter-e task would not deplete self-regulatory resources as participants did not have to overcome an habitual response, which they argue is a necessary requirement to induce depletion. Consequently, they argued that the lack of a depletion effect in the outcome task was understandable as the treatment task was not ego-depleting, because of the way in which the task was administered.

### The Need for a New Type of Evidence in Ego-Depletion

While evidence for the ego-depletion effect remains inconclusive, it is unlikely that mere replications will be sufficient to properly evaluate the strength model. Lurquin and Miyake (2017) comment that to overcome “the conceptual crisis for the ego-depletion literature” (p. 1) performance on the intervention task needs to be independently evaluated. In other words, a major omission in the ego-depletion literature, and within the sequential task-paradigm specifically, is that performance on the intervention task is rarely examined and more importantly has not been shown to produce a decrement in performance that would be indicative of a resource depletion. This lack is partly due to the fact that many of the intervention tasks that have been adopted (e.g., not thinking of a white bear) are not readily amenable to measurement. However, until it can be demonstrated that intervention tasks do result in depleted resources, a key theoretical assumption of the strength model will remain just that, an assumption.

One popular depletion induction task where performance can be tracked is the letter-crossing task. The letter-crossing task is one of the most commonly used intervention tasks (Hagger et al., 2010; Carter et al., 2015) and is a type of search and identification task. It involves participants locating a particular letter, generally the letter *e*, according to different sets of rules. The most common operation is as follows. Participants are asked to cross out every letter *e* on a page of text, the point being to establish a habitual response. Once completed, in the experimental condition participants are given additional pages of text and additional rules that contravene the habitual response. For example, the first rule might instruct participants to cross out an *e* in cases where there is another vowel immediately before or after it. A second rule typically accompanies this rule and instructs them not to cross out the *e* if the vowel before or after is an *i*. Effectively, this means participants respond to *ae, ea, ee, eo, oe, eu*, and *ue* combinations, but *inhibit any response to ei* or *ie*. In the control condition, participants’ may simply be asked to continue with the same rule as for the first page and cross out every letter *e* on the following pages. The theoretical rationale for why the experimental condition consumes more self-regulatory resources than the control is straightforward. The instructions of the first page instill a habit (i.e., cross out every letter *e*) and the instructions on the second page force the participants to inhibit this habituated response (i.e., do not cross out the *e* in specific cases). It is argued that the overriding of this newly acquired habitual response is the cause of resource-depletion (Tice et al., 2007; Wheeler et al., 2007; Clarkson et al., 2010; DeWall et al., 2011; Boucher and Kofos, 2012; Molden et al., 2012; Salmon et al., 2014; Xu et al., 2014; Achtziger et al., 2015; Chow et al., 2015; Harkness et al., 2015; Petrocelli et al., 2015; Wang et al., 2015; Baumeister and Vohs, 2016; Haynes et al., 2016; Jia and Hirt, 2016; Voce and Moston, 2016).

The pertinent point for the present discussion is that, using this common task, both the experimental and control conditions require behavioral responses that can be tracked over time. This is critical since the depletion of regulatory resources is a within-subjects effect (occurs within participants over time) and the standard approach to observing it has been between-subjects using comparisons of experimental conditions. Currently, the only investigation known to the authors that systematically measured change across time is reported by Arber et al. (2017). In this study, participants were presented with five written stories (one per page as each story represented a page of text) and, in the experimental condition, were required to follow the rules just described regarding responses to vowel pairs. Across five independent studies, participants’ ability to detect target vowel combinations did in fact decline as a function of time on task (i.e., as they completed five stories/pages; see **Figure 2**). Furthermore, for those participants that showed performance decrements across time, deterioration in performance was also observed on a secondary outcome task (working memory span). At face value, finding a time dependent decline on the experimental task with carry-over effects on the outcome task is totally consistent with the predictions of the strength model. However, there are some aspects of that study that are problematic.

Rather than having an active control where participants identify instances of the letter *e*, Arber et al. (2017) utilized a passive control condition, in that they had a 10-min (equal to the experimental depletion-induction task length) “chat” with the experimenter before doing the outcome task. As such, it is yet to be shown whether the commonly used active control procedure would or would not show the same negative performance gradient. Finding an equivalent negative gradient of the control task would be particularly problematic for the strength model. Further, the procedures used by Arber et al. (2017) did not have the habit forming first page. All pages required the application of the two vowel pair rules. The researchers argued that having participants identify the letter *e* in one set of cases and not in another set of cases would be sufficient to cause resource depletion, as this is a self-regulatory action that would require resources. This notion is not without precedence; other researchers have also argued that this immediate implantation of both rules would be sufficient to cause ego-depletion (Baumeister et al., 1998; Fischer et al., 2007; Wan and Sternthal, 2008; Halali et al., 2014; Sripada et al., 2014; Hagger and Chatzisarantis, 2016).

This presence or absence of a habit-forming component in the letter-crossing task, however, has been the cause of recent debate, with Baumeister and Vohs (2016) listing the lack of a habit-forming stage in the Hagger and Chatzisarantis (2016) multi-lab replication study as a reason to dismiss the null findings. Baumeister and Vohs comment that the replications study’s version of the letter-crossing task, which also did not start with a habit forming stage prior to the instigation of a new set of rules, was an essential methodological flaw that invalidated the non-effects on the outcome measure. “Without first instilling the habit, there is nothing to override. This may be a difficult cognitive judgment task, but no impulse is overridden, contrary to the nature of self-control tasks” (Baumeister and Vohs, 2016, p. 574). Although there have been theoretical justifications for the requirement of behavioral inhibition within the letter-crossing task (e.g., Baumeister and Vohs, 2016), there has yet to be independent empirical evidence to give credence to such justifications. This dispute between the leading researchers as to whether a treatment condition is, in fact, ego-depleting, further emphasizes the need to empirically justify the theoretical claims made within the ego-depletion literature as Lurquin and Miyake (2017) have recommended.

Although the Arber et al. (2017) findings argue against the need for a habit-forming stage, from an empirical standpoint, the question remains as to whether the request to inhibit an *ie* or *ei* response is the cause of the decrement in performance in their data as alternative explanations could explain the negative performance gradient they found. For instance, it could be argued that the vowel pairs are embedded in text and that the participants must override an automatic response to read the words presented in the stimuli when they are trying to engage in the primary task of locating specific letters. While this explanation is acceptable within the limited-strength model, it is not the justification given within the literature. Additionally, if this alternative explanation can account for the depleting effect, then it would be equal across experimental and control conditions as standardly conceived. That is, both the control condition and the experimental conditions both require the overriding of the automatic reading process. Alternatively, simply following multiple rules could be the cause of depletion by merely increasing cognitive load (increasing task difficulty), and the fact that the second rule involves behavioral inhibition is only incidental and not necessary contributing to the depletion effects.

One might argue that the above manipulations simply increase the complexity and difficulty of the letter-e task. While manipulations of task difficulty across different presentations might serve to reduce performance (obviously, a more difficult task leads to poorer performance), task difficulty alone can’t account for the trend of declining performance with time on task. A more difficult task will mean poorer performance (a main effect in a sense) when compared to a less difficult task but it won’t necessarily produce greater deficits in performance over time. If task difficulty were the only process accounting for performance variance, we might expect the degree of decrement in performance over time to remain constant (or even improve due to practice effects) regardless of the difficulty of the task – though overall performance will be poorer when compared to an easier task. This is partly what supports our inference that performance decrements in a task like this may reflect declining resources available to do the task.

In general the letter e task is fairly easy (it is an effortful task but not a difficult task) and that is partly why we don’t observe practice effects like we do with more difficult skill-based tasks like Stroop tasks (people get better at it over time and therefore performance is enhanced). Difficult tasks plausibly require greater skill acquisition and practice to maximize ability, whereas the letter e task does not require much skill to complete. We believe characteristics of the task make it ideal for investigations into resource depletion.

Task difficulty across our experiments can plausibly account for variation in the overall performance differences between participants but cannot solely account for declining performance over time if the difficulty of the task remains constant through administration (i.e., over time). The task becomes more difficult for the participant over time as their resources decline, though the actual demands of the task remain constant.

### The Present Studies

It is the goal of the following investigation to understand the factors that drive performance on the letter-e task. The first experiment was designed to explicitly test the Baumeister and Vohs (2016) assertion that habit forming and subsequent inhibition of that habit are necessary for resource depletion to occur. The key variable is the accuracy of target detection, and we assume that the negative performance gradient (see **Figure 2**) reported by Arber et al. (2017) will change as a function of the manipulations introduced. To preview our results, we could find little evidence in support of these assumptions or for the strength model. The strength model as it is currently articulated, could not provide a compelling theoretical basis for performance on the letter-e task.

As mentioned earlier Arber et al. (2017) did present empirical data that demonstrated a decline over time consistent with the notions of resource depletion. The follow-up experiments were designed to be an in-depth, exploratory examination of the stimuli and procedures used in their experiments. The intent here was to examine whether or not the marked decrement in performance could be the result of possible confounds in linguistic features of the materials they used, or the fact that the materials all involved coherent text.

## Experiment 1

The first experiment is a conceptual replication of the Arber et al. (2017) study with three procedural differences which include (a) using quasi-random letters for the test materials instead of coherent text, (b) using a habit formation stage in the depleting condition as suggested by Baumeister and Vohs (2016), and (c) using three active conditions—one control group and two treatment groups.

The current study uses quasi-random letters as the stimuli to eliminate any automatic reading response as an alternative explanation for any decrement in performance. The text is quasi-random because the number of target items remains constant across pages and conditions, but their location is randomized within each page. Additionally, like the Arber et al. (2017) study, this experiment tracks participants’ performance across five sections of text, but unlike Arber et al. (2017), and in line with Baumeister and Vohs (2016) mandate, this experiment has one page for habit-forming followed by four pages in which the participants have to override this habitual response.

The final difference involves the experimental groups in the study. Unlike the Arber et al. (2017) study, the current experiment adopts an active control condition. Participants will be asked to circle every letter *e* they can locate and this rule will not change over the five pages of text. These participants will not be affected by any rules that require behavioral inhibition or affected by the requirement of following multiple rules.

This study utilizes two treatment conditions, the first of which we have labeled the inhibition condition. In this condition, participants circle all *e*s (i.e., any letter e that has an *a*, *e*, *i, o,* or *u* directly before or after it) on the first page. Then on the following four pages, they are asked to continue to circle *e*-vowel pairs except if that pair contains an *i* (i.e., *ei* or *ie* pairs). In these cases, they are to refrain from circling the *e*-vowel pairs. In this manner, and in line with Baumeister and Vohs (2016) argument and previous justifications (e.g., Tice et al., 2007; DeWall et al., 2011), the participants in this condition will have one page in which they form a habit (i.e., circling every *e*-vowel pair) followed by four pages in which they will have to override this response (i.e., not circling the *e*-vowel pairs that contain the letter *i*). According to the limited-strength model, because this task involves repeatedly overriding a habitual response, a decrease in performance across the pages of text is hypothesized.

The other treatment condition we have labeled the no-inhibition condition. Participants start with one page in which they circle every *e*-vowel pair (i.e., any *e* that has an *a*, *e*, *i*, *o*, or *u* immediately before or after it). This is followed by four more pages on which they continue to circle *e*-vowel pairs. However, for these final four pages, there is an additional rule for *e*-vowel pairs that contain the letter *i*. That is in these cases, and in contrast to the inhibition condition, participants must circle *and* underline *e*-vowel pairs that contain the letter *i*. These participants are thus asked to detect *ei* and *ie* pairs, and to produce an alternate response, not inhibit a response. Although this task is as computationally difficult in the same way as the inhibition condition, according to the key premise that been the subject of recent debate, this task should not deplete self-regulatory resources as no behavioral inhibition is required (Baumeister and Vohs, 2016) and no decrement in performance across pages should be observed. However, if performance does deteriorate in the no-inhibition condition, this would suggest that following multiple rules is necessary for the effect and not behavioral inhibition *per se*.

In sum, the specific expectations derived from the strength model perspective are that there will be no significant difference in accuracy rates on the first page of text between the three conditions, as the behaviors only change after the completion of the first page of text. This will serve as the pre-test to check group equivalence. If Baumeister and Vohs (2016) are correct in their assertions, the accuracy rate will significantly decline across the final four pages of text for the inhibition condition, but no significant differences will be seen across these pages in the no-inhibition and control conditions.

### Method

#### Ethics Statement

This study was carried out in accordance with the recommendations of NHMRC National Statement on Ethical Conduct in Human Research (2007) that governs research involving human participants in Australia. All subjects gave written informed consent in accordance with the Declaration of Helsinki. The protocol was approved by the Human Research Ethics Committee of the University of Southern Queensland.

#### Participants

The total sample size for the experiment was *N* = 77; however, data from three participants were removed because they did not follow their conditions instructions. The remaining participants’ age ranged from 18 to 74 years old with an average of 31.49 years (*SD* = 11.39 years), 48% were females, and 70% were studying at University or had university degrees. All participants for the experiment needed to be 18 years of age or older, have English as their predominant language and read basic English. While all participation for the experiment was voluntary and was conveniently sampled, a small portion (*n* = 6) were 1st-year psychology students who received course credit for participating in the experiment. All other participants were recruited from the researcher’s social and professional networks and received no incentive to participate in the experiment.

#### Materials

**FIGURE 1:**
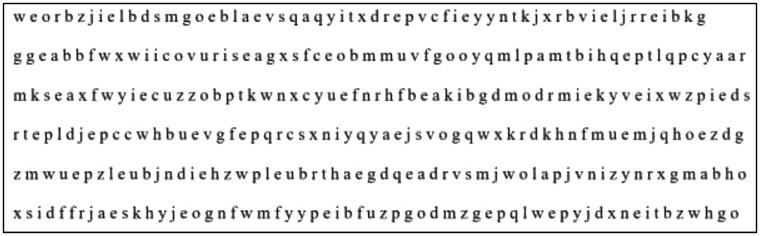
Excerpt of test material for the letter-crossing task of the current experiment. This excerpt was from the control condition so all cases of “e e” that are present in the other two conditions have been replaced with “e p.”

The study booklet consisted of seven pages of materials, of which the first five pages were used. The final two pages were dummy pages to ensure that participants did not anticipate the end of the task. The seven pages of test material were printed on white A4 paper with black, size eight, Times New Roman font with double line spacing. Each page consisted of 1,800 letters with a single space between each letter. There were 60 characters per line and 30 lines per page. On the five critical pages, each page contained 200 letter *e*s, with a vowel directly before or after each *e* (see **Figure 1** for an excerpt of a page of test materials). In the test pages for participants’ in the control condition, every instance of a double *e* was replaced with an *e p*. This was done so the location of the targets remained constant across conditions but ensured that each page only consisted of 200 targets; as a double *e* in this condition would be considered as two separate targets but that same double *e* would be considered as one target in both treatment conditions.

Aside from this change, the only difference in test materials between conditions was the printed instructions given to the participants. As such, each participant had 200 targets to identify on each page. As the control condition and the first page of both treatment conditions only had the one rule, all 200 targets on each of these pages corresponded to that one rule. On the remaining four pages in the treatment conditions, each page consisted of 120 targets that corresponded to the treatment conditions’ first rule (*e* plus *a*, *e*, *o*, *u*), and 80 that corresponded the second rule (*e i* or *i e* combinations).

To ensure that all pages consisted of 200 target items, with their location randomly distributed within each page, the 1,800 characters were comprised of 300 six-letter strings. The strings that did not contain any targets were made of random letters generated from a list of all alphabet letters—with replacement—excluding the letter *e*. For those strings that did contain a target, the letter *e* was located in one of four internal serial positions with a consonant always residing in the first and sixth serial position. The consequence of this is that target items could vary in position within the letter string but target items could not interact with one another between letter strings because there are always at least two consonants between them. In total, each page contained 200 letter-string that contained vowel combinations and 100 letter-strings that contained no target items. This ratio was consistent across the five pages; however, new letter-strings were generated for each page and a new random order of letter-strings was generated for each page. Only after the letter-strings and ordering were generated were the double *e*s in the control condition replaced with the letters *e* and *p*.

The dependent measure in the control condition was the proportion of letter *e*s detected by the participants. For both the inhibition and no-inhibition conditions, the DV was the number of *e*-vowel combinations detected on the first page, but on the remaining four pages, it was the number of *e*-vowel combinations that involved the letters *a*, *e*, *o*, and *u*. This dependent measure was chosen for two reasons. Firstly, Arber et al. (2017) showed a reliable decline in the accuracy rate of this measure across time as the strength model would predict and, for those who showed self-regulatory errors on the secondary rule, this decline was positively correlated with performance on a secondary task (again, consistent with the strength model). Secondly, it is the only measurable outcome that does not lend itself to contradicting interpretations. For instance, spending more time on each page could be indicative of both having greater self-regulatory resources allowing participants to persist longer (as in Baumeister et al., 1998, Experiment 1), and as having less self-regulatory resources resulting in slower reaction times (see Hagger and Chatzisarantis, 2016) for each item on the page. Also, random errors per page also lead to contradicting conclusions with random errors being indicative of low self-regulatory resources resulting in failures to correctly follow the instructions, as well as no random errors being interpreted as having lower self-regulatory resources resulting in an increased passive-option effect (see Baumeister et al., 1998, Experiment 4) making random errors actually less likely. Finally, tracking performance on targets that correspond with the second rule (i.e., *e*-vowel pairs that contain the letter *i*) becomes uninterpretable for the inhibition condition. This because they are told to not circle these vowel pairs making it impossible to know if they are correctly applying the rules to this target or are simply missing these targets.

#### Procedures

Following written, informed consent, each participant was tested individually. Participants were allocated to one of the three experimental conditions in a pseudo-random manner, such that there were at least 25 participants in each condition. All participants within a condition given the same order of the five pages of the test materials. Those in the control condition were first given their written instructions that told them to circle every letter *e* they could find and they were then given an example line to practice. After correct completion of this practice line, they were given the remaining pages of the test materials.

For both treatment conditions, participants were first given the same set of instructions that directed them to circle every letter *e* that had a vowel (*a*, *e*, *i*, *o,* or u) directly before or after it. They were also given a test line to ensure they understood their instructions, followed by the first page of test materials. After the participants completed their first page of text, those in the inhibition condition were given their new set of instructions that directed them to continue circling *e*-vowel pairs except if that pair contained an *i* (e.g., *ei* or *ie*). In those cases, they were told to *not circle* the pair. Those in the no-inhibition condition were also told to continue circling e-vowel pairs. They were also given an additional rule that directed them to *circle and underline* any *e* that had an *i* directly before or after it (e.g., *e i* or *i e*). Participants in both treatment conditions were given example lines to ensure they understood their new instructions. After they correctly completed this example line, they were then given the remaining four pages of text to complete. All participants were told that both their speed (which was recorded for each page of text) and accuracy were being recorded, and to work from top-to-bottom and from left-to-right as if they were reading the text. After the participants had completed the task, they were debriefed and thanked for their participation. The materials were then marked for accuracy. For each page, a proportion of target items correctly identified by the participant for each corresponding rule was calculated. Instances of random errors (i.e., circling letters did not comply with any rule) were also counted for each page of text.

#### Data Analysis Plan

All data for the following experiments can be found at https://osf.io/rnz5w/. All four experiments reported here deal with a single measure – the number of target items correctly detected. While other measures are possible, we have limited our focus to target identification because it is an obvious measure to test the assumptions of the strength model and empirically it has been shown to be sensitive to time on task and carry-over effects have been observed based on this measure (Arber et al., 2017). The means of all conditions in each experiment are reported, although not necessarily analyzed in all experiments. Sampling procedures are described in each method section which make it clear that sample size was determined prior to the commencement of testing and was determined by the limited time available to recruit participants. Decisions regarding the relationship between sample size and power are described in the following paragraph for Experiment 1 and the results section of Experiment 2. Data from all participants were used, unless indicated otherwise (Experiment 1) and data were not inspected until all participants had been tested.

Since the hypotheses of the experiment center on the presence or absence of an interaction between condition and time on task (story number), two *a priori* power analyses using G^∗^power were conducted to evaluate the minimum sample size needed to detect that interaction. This analysis first established the sample size needed to detect an effect size equivalent to the large effect obtained in Arber et al. (2017). The second power analysis was conducted to determine the number of participants needed to detect a more conservative medium effect size. With the error rate set to 0.05, power set to 0.95 and a correlation of 0.5 among repeated measures a total sample of 39 was sufficient to test the critical interaction for both large and medium effect sizes. We did not use this power analysis as a stopping rule for determining the final number of participants, preferring to use samples sizes that were more indicative of prior studies. Consequently, the current sample exceeds that required to adequately evaluate the critical interaction.

All null-hypothesis significance testing was conducted using SPSS (version 20) with an α-level set at 0.05 (two-tailed). In addition to null-hypothesis testing, supplemental Bayesian statistics were conducted using JASP (version 0.8.1.2). The use of Bayesian statistics, and subsequent calculation of Bayes Factors (BF), assesses (a) evidence for the alternate hypothesis, (b) evidence for the null hypothesis and (c) no evidence for one over the other (Dienes, 2011), where the importance of experimental power is diminished. For example, a *BF*_01_ = 11, indicates that the data is 11 times more likely under the null hypothesis than the alternate hypothesis. Note that the sub-script of 01 is an indication of reference in favor of the null hypothesis. If the sub-script was *BF*_10_ this would then be interpreted as the data being 11 times more likely under the alternate hypothesis than the null hypothesis (Wagenmakers et al., 2017). BF of 3 and above are considered to be evidence for that specific hypothesis denoted by the subscript (Wagenmakers et al., 2017). The calculation for Bayesian statistics does require a prior distribution to be set–*a prior* belief as to what the effect is–and for the following analysis, the uninformed priors recommended by Wagenmakers et al. (2017) were utilized.

### Results

**FIGURE 2:**
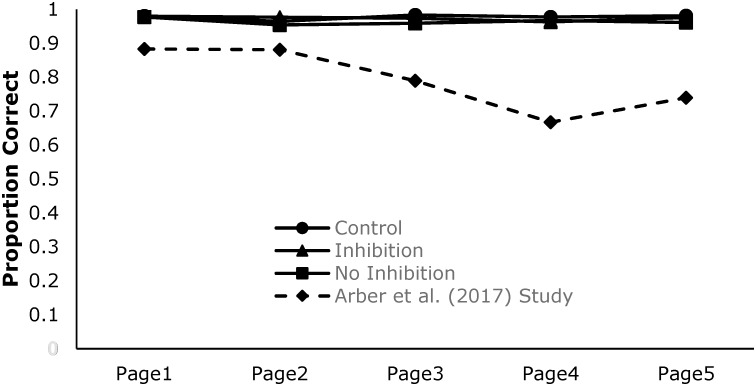
Accuracy rate of the participants for the first rule of five pages of text. The dashed line is the result of previous research conducted by Arber et al. (2017). One page of text for the current study and one story for Arber et al. (2017) study represent functionally equivalent units of text.

**Figure 2** shows participants accuracy rates over the five pages of text for the three conditions (Control *n* = 27, Inhibition *n =* 23, and No-Inhibition *n* = 24). It also displays the results of one of the studies from the aforementioned Arber et al. (2017) article for a visual comparison of a previously found negative gradient. As can be seen in **Figure 2**, participants’ accuracy rates were markedly similar on the first page of text, indicating that the groups were equivalent at the beginning of the task. Despite this, the obtained results differ markedly from Arber et al.’s previous findings, in that there is little deterioration across pages, and little difference among the three conditions.

To test for differences in accuracy rates for the first page of text, a one-way between-subjects ANOVA was conducted with the three levels of the experimental condition set as the fixed-factor and accuracy of the first page set as the DV. The results suggested that there was no difference between the groups for the first page of text, *F*(2,69) = 0.14, *p* = 0.87, $\eta_{p}^{2}$ < 0.01, *BF*_01_ = 7.48.

To test for differences in accuracy rates on the following four pages a 3(condition) × 4(page [2–5]) mixed factorial ANOVA was conducted with accuracy as the DV. There was no main effect of accuracy rates across the four pages of text, *F*(3,213) = 0.52, *p* = 0.67, $\eta_{p}^{2}$ = 0.01, *BF*_01_ = 35.82, however, for the main effect of experimental condition significant differences in accuracy rates were found, *F*_w_(2,44.86) = 5.11, *p* = 0.010, $\eta_{p}^{2}$ = 0.19, *BF*_10_ = 12.78. Furthermore, the condition × page interaction was significant, *F*(6,213) = 2.40, *p* = 0.029, $\eta_{p}^{2}$ = 0.06, *BF*_01_ = 1.13.

To examine the condition × page interaction a one-way repeated measures ANOVA was conducted for each of the three experimental conditions. The results indicated that there was no significant change in performance for the control condition, *F*_G_(1.77, 46.05) = 0.87, *p* = 0.46, $\eta_{p}^{2}$ = 0.03, *BF*_01_ = 7.59, and the no-inhibition condition, *F*(3,69) = 1.43, *p* = 0.24, $\eta_{p}^{2}$ = 0.06, *BF*_01_ = 3.76, with evidence supporting the null hypothesis in both conditions. While, the page effect for the inhibition condition was also non-significant using a strict 0.05 cut-off, *F*(3,66) = 2.56, *p* = 0.06, $\eta_{p}^{2}$ = 0.10, *BF*_01_ = 1.03, the *p*-value approached significance. However, the Bayesian analysis favored neither the null nor alternate hypotheses.

### Discussion

The first motivation of the current study was a general criticism of the resource-depletion literature. Specifically, that there has been little demonstration that the depletion tasks employed in the sequential-task paradigm actually resulted in decrements in performance consistent with a depleting pool of self-regulatory resources. The second, and more specific motivation, was based on Baumeister and Vohs (2016) assertion that depletion only occurs under a limited set of conditions; namely that a habit formation stage was necessary and that response inhibition of this habit was the causal factor in producing depletion effects. Consequently, it was expected that all groups would be equivalent on the first page of text when no response inhibition was required, but they should differ on subsequent pages. Specifically, it was expected that there would be no deterioration in performance across pages for those in the control condition and the no-inhibition condition as neither condition involved overriding a habitual response and therefore should not deplete self-regulatory resources. It was, however, expected that those in the inhibition condition would deplete self-regulatory resources which would be reflected in a performance decrement similar to that observed by Arber et al. (2017).

The outcomes of the study met all but one expectation. As predicted, the three groups were equivalent on the first page of text. Additionally, the control and no-inhibition conditions did not show any decrement in performance consistent with the notion that self-regulatory resources were not depleted, and that depletion may require habit-formation and dominant response-inhibition. However, contrary to model-based expectations, participants in the inhibition condition also did not show a marked decrement in performance across pages, and certainly not of the magnitude that was observed in the Arber et al. (2017) experiments. In short, there was no compelling evidence for performance deficits in any of the three conditions.

Counter to Baumeister and Vohs (2016) position, the results of this study do not support the notion that the behavioral inhibition implemented within the letter-crossing task drives the resource-depletion effect. The crucial decline in accuracy rates across page, which would suggest that self-regulatory resources were depleting, was not witnessed. The current data exhibited a trend in the expected direction but the effect was non-significant and the magnitude of the effect was unlike that observed in prior studies.

Given that the inhibition instructions were practically identical to those used in the Arber et al. (2017) study, a potential cause for the different outcomes in the magnitude of deterioration may be the stimuli used. Specifically, the Arber et al. (2017) study used intact stories for their stimuli, thereby containing a narrative to be read, while the current study used lines of random letters, thereby containing no elements that could be read. Given that it is widely understood that a large component of reading is automatic (Walczyk, 2000), it could be argued that the participants within the Arber et al. (2017) study had to override their automatic response to read the text when they were complying with the rules that were given to them. Furthermore, it is plausible that it was this overriding of their automatic reading response that was the cause of Arber et al.’s negative performance gradient. This would be in contrast to the current study in which there was no automatic reading response to overcome potentially contributing to the lack of resource-depletion. In short, the comparison of the effects observed between the studies would suggest that while participants engage in a secondary task (i.e., following the multiple rules of the letter-crossing task) some aspect of readable language in the stimuli is the active element needed to induce the resource-depletion effect.

The other major difference from the Arber et al. (2017) study was that in the current study, every letter *e* was paired with a vowel and therefore constituted a target. This is opposed to the Arber et al. (2017) materials in which isolated *e*s were present, meaning that there were *e*s that were not a target. Although this latter case is more reflective of the letter-crossing tasks that have been historically used, it does lead to an interesting question. That is, what habits are being overridden in the letter-crossing task? While researchers posit on theoretical grounds that participants form a habit of circling *e*-vowel pairs, operationally participants are historically exposed to more isolated *e*s than *e*-vowel pairs. It could then be argued that the first habit formed would come from not circling isolated *e*s which then must be broken to circle the *e*-vowel pairs, which then again must be broken if that vowel pair complies with the secondary *ei*, *ie* rule. This three-stage process present in the Arber et al. (2017) study was not employed within the current study and its absence may have contributed to the null finding. In other words, having multiple levels to the self-regulatory task may have additive or multiplicative effects on depleting self-regulatory resources and only having a two-stage self-regulatory process in the current study may have massively reduced the resource-depletion effect.

A third difference involves the fact that the letter-e task was presented in pencil and paper format. Arber et al. (2017) tested both pencil and paper and computer presented versions of the letter-e task, with all conditions producing negative performance gradients, so presentation format was not expected to influence the current results. However, to confirm this assumption, the remaining experiments incorporate computer presentation of text with participants verbally identifying the target items.

While the changes made to the presentation format were incorporated to control for language factors, it is possible that the changes have fundamentally changed the task. In fact, the stimuli in the experiment are very similar to those used in the Mesulam-Weintraub Cancellation Test (Mesulam, 1985), a standardized test that is used in neuropsychological assessment of visual scanning speed and visual neglect. The first trial on the test consists of a page containing rows/columns of random letters almost identical to those displayed in **Figure 1**, and the task is to search for the letter A in each of the rows/columns. These authors reported that normal adults could complete each of the four tests without error in less than 2 min. The fact that in the current experiment performance is also virtually error free, suggests the possibility that we have fundamentally changed the task turning it into a visual search task rather than a test of resources required for self-regulation. If this is so, then this test does not provide a fair test of the strength model.

To this point we have assumed that the results of Experiment 1 are somehow aberrant or do not provide a fair test of the strength model. It is, however, possible that the Arber et al. (2017) results are the aberrant findings. The materials in their study, five short stories, were sourced from the internet without any thought to the characteristics of the selected text. Moreover, the stories were always presented in the same order. Thus, if there was factor that made some targets more difficult to detect than others, and that was more prevalent in later stories than earlier stories, then deterioration in target detection across time could emerge. The possibility remains that confounds could be the causal factor for the decrement rather than depletion of self-regulatory resources. Alternatively, if the current test is not a fair test of the strength model, ithas nothing to say about the depletion process that is apparent in the Arber et al. (2017) findings. On both counts, a much closer inspection of the materials used in the Arber et al. (2017) experiment appear to be warranted.

One possible factor that may be playing a role in target identification is bigram frequency (i.e., the frequency of two-letter combinations occurring in the language). It is known that the frequency that the different e-vowel combinations occur in the written language have an impact on many cognitive tasks. In the visual word identification literature, for example, it is well established that bigram frequency has an impact upon reading and word identification, with frequently occurring combinations being associated with better performance [see Chetail (2015), for a review]. We took as a starting point, that identification of targets in coherent text would be related to the bigram frequency of the vowel combinations, with targets containing frequently occurring combinations like *ea* being better detected than targets containing rarer combinations like *ae.* If such an outcome was obtained, the interest then centered on how those differences changed across stories. It should be noted that such an exploration requires that we measure the degree to which specific words in the text were identified.

We were also interested in testing the notion that the discrepancies in the magnitude of the deterioration over time between the Arber et al. (2017) and Experiment 1 results were related to the interference that reading coherent prose produces. As such, we adopt the materials and procedures that Arber et al. (2017) employed (Experiment 2), but we manipulate the intelligibility of the prose by randomly re-ordering the words in each story (Experiment 3) or randomizing the letters in each word in each story (Experiment 4), while retaining the same surface structure (punctuation, sentence, and paragraph structure), with the target vowels being maintained in the same location across stories in all three experiments. Here we were interested in the extent to which absolute levels of performance would increase, and how bigram frequency effects might vary with the change in coherence.

## Experiment 2

In exploring the impact of bigram frequency on target detection in the letter-e task, we utilize the materials of Arber et al. (2017). In that study, five short passages of text with 115 target items and 24 *ie* and *ei* words were used. In this and following experiments the independent variable was the specific vowel combinations, of which there were seven different combinations (see **Table 1**). However, in the statistical analyses, we have limited the conditions to the two most frequently occurring combinations *ea* (most frequent) and *ee* (less frequent), primarily because these are the only two conditions where there are examples in all five stories. The dependent variable in all studies was the proportion of participants who identified the target item. If bigram frequency has an effect upon performance then the expectation would be that targets containing frequently occurring combinations will be better recalled than less frequent combinations. It is also expected that target detection should deteriorate across stories.

**Table 1:** Characteristics of the stories used in Experiments 2–4.

	Story 1	Story 2	Story 3	Story 4	Story 5	Total	Normative
Words	249	294	307	535	555	1940	
Characters	1105	1210	1237	2217	2457	8226	
Single e	107	134	109	215	260	825	
Targets	19	13	23	20	39	115	

ae				1		1	0.3
ea	15	7	13	11	21	67	19.4
ee	4	6	9	8	7	34	10.6
oe				1	3	4	1
eo			1		4	5	2
ue					3	3	4.1
eu					1	1	0.8

ie		4	4	2	5	15	10.8
ei		2		3	4	9	5.1

*Normative values are in billions (e.g., 19.4 billion words in the corpus contain the ea combination.*

### Method

#### Participants

One-hundred and thirty-two people participated in the experiment. Recruitment of participants was a course requirement for 22 students enrolled in a class devoted to developing practical research skills. Each student was asked to recruit 6 participants from their social network, and where possible ensure equal numbers of male and female participants, and a large age range. It was a sample of convenience that was derived from the wider community. Females made up 58% of the sample, age ranged from 18 to 90 years with a mean of 42.0 years (*SD* = 20.0), with 65% not having studied at university.

#### Materials

**FIGURE 3:**
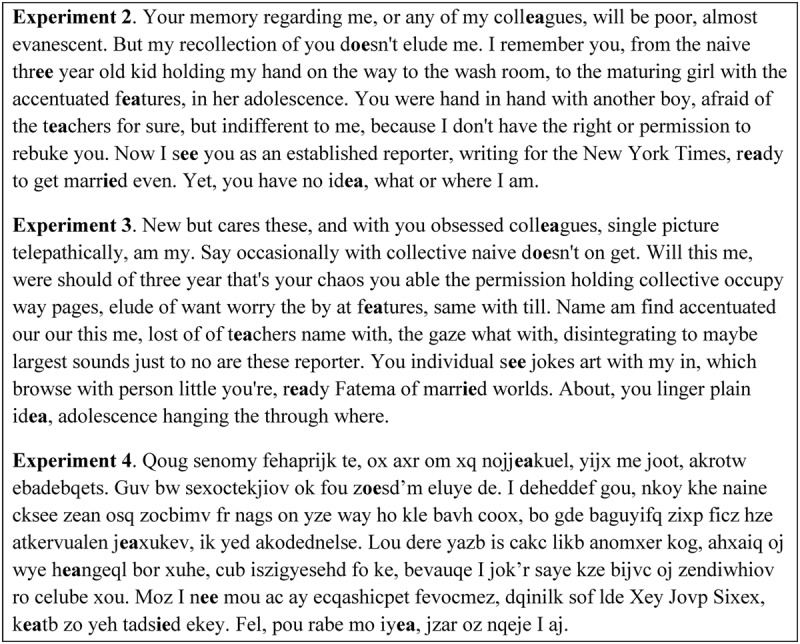
Sample materials used in Experiments 2, 3, and 4. Targets and distractors are presented in bold font in this example, but not in the text that participants viewed.

In this experiment we used the coherent stories of Arber et al. (2017). **Figure 3** shows an example of one paragraph from one story for each of the subsequent experiments.

As mentioned earlier, the five stories were sourced from the internet with little consideration as to the content or linguistic properties of the material. Each of the stories conformed to standard textual constraints in that the words were organized into sentences containing standard punctuation requirements, and the sentences were organized into paragraphs. In **Table 1**, we present the characteristics of each story, with particular emphasis on the distribution of e-vowel pairs in each story. The first point of note is that the ratio of single *e*’s to e-vowel pairs is 5.97:1. Secondly, *ea* and *ee* pairs are the most frequent pairs overall, with the other rarer combinations more frequent in the last two stories than in the first three stories. In the right hand columns of the table, we compare the distribution of the specific vowel combinations with that found in the Google books corpus (Norvig, 2013). The rank order correlation between the frequency distribution obtained in our materials with that in the large corpus was 0.95. Thus, the stories we use appear to be a good sample of a much larger corpus.

#### Procedure

As per Arber et al. (2017). Each story was presented in its entirety on a single slide as on a PowerPoint presentation. The five stories/slides were presented in the same order for all participants. Unlike Experiment 1, the first page was not a habit-forming page. The rules of responding to *e* words that were preceded by or followed by either an *a, e, o,* or *u*, but to not respond if the vowel was an *i*, applied throughout the five stories. Practice at using these rules was provided prior to the presentation of each story. The instructions were for participants to read the stories as quickly as they could and to verbally identify each target item by saying it aloud. Once a story had been completed, participants pressed the space bar on the computer to advance to the next story/slide. For each story the researcher recorded whether or not each target was identified, whether any of the ie/ei words were identified, and they recorded the time to complete the story.

### Results and Discussion

The current analysis in this and subsequent experiments are based on 67 *ea* targets and the 34 *ee* targets that were present in the five stories. Given that the number of items is predetermined our power analysis was necessarily *post hoc* and was used to determine the size of the effect that could be detected with this sample size. The outcomes indicated that we could detect medium to large effects sizes for bigram frequency effects, small to medium effect sizes for deterioration across stories, and small to medium effect sizes for the interaction.

**FIGURE 4:**
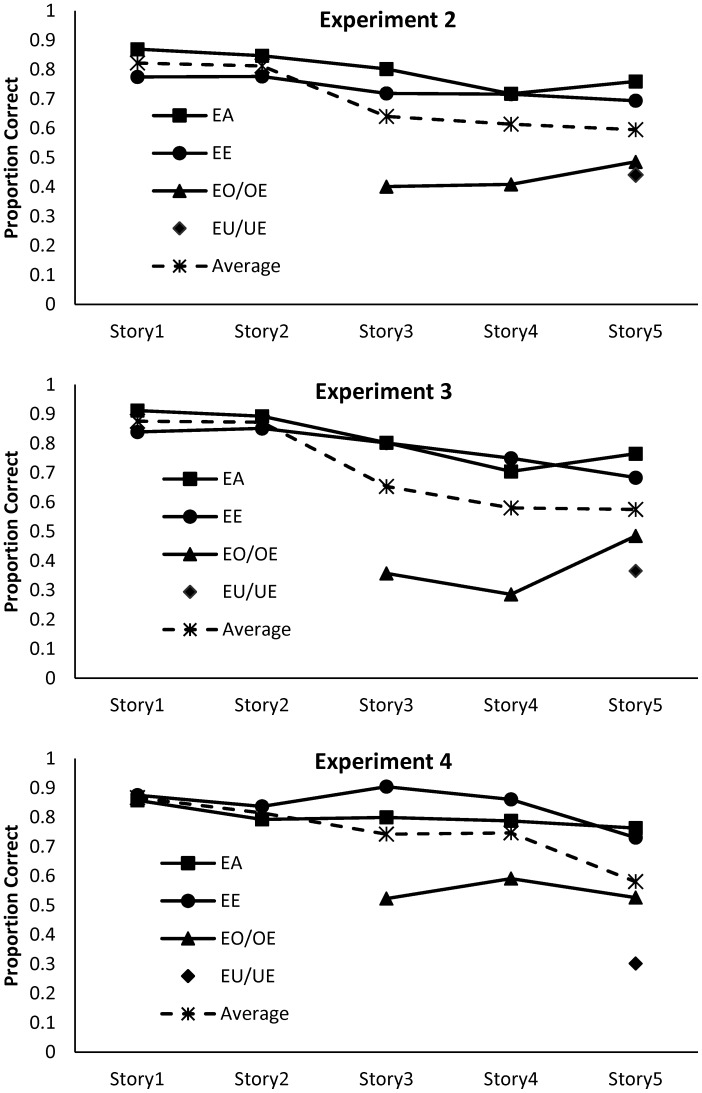
Target detection in Experiments 2, 3, and 4 as a function of vowel combination and story. The average of all conditions is represented by the dotted line.

The results of the experiment are summarized in **Figure 4**, where the dependent variable is the proportion of participants who correctly identified the target items. There are three patterns that emerged: target recognition differed considerably for the specific e-vowel combinations; for the frequently occurring combinations, performance deteriorated across stories; and, the rate of decline across stories was the same for the frequently occurring (*ea* and *ee*) combinations. With regards to the specific combinations, *ea* and *ee* combinations were reasonably well recalled but combinations involving *o* and *u* were not well identified. This outcome suggests that there is a close link between target identification and bigram frequency when participants are searching for specific letter combinations in coherent text. Additionally, the poor identification of infrequent combinations in Stories 3–5 suggests that the decrement in performance observed for these stories in the Arber et al. (2017) experiments were due, in part, to a combination of vowel combinations and story.

In order to determine the degree of decline in target detection for the high frequency targets, a 2 × 5 mixed factorial ANOVA was conducted with vowel type *(ea* versus *ee*) as the first factor and the five stories being the second factor. The outcomes of the analyses and Bayesian evidence in favor of the experimental hypothesis are presented in **Table 2**. The two main effects were significant. Target detection was more accurate for *ea* combinations than *ee* combinations, and performance did deteriorate across stories, with performance differing significantly from Story 1 to Story 4. There was no significant interaction, with the Bayesian analysis indicating strong evidence in favor of the null hypothesis.

**Table 2:** ANOVA main effects and interactions, Bayes Factor, and point at which performance was below Story 1, for Experiments 2, 3, and 4.

	*F*	*p*	$\eta_{p}^{2}$	BF_10_	Decrement
**Experiment 2**					
Story	3.24	0.016	0.13	23.04	Story 4
Vowel	7.49	0.007	0.08	6.08	
Story × Vowel	0.52	0.718	0.02	0.19 (4.71)	
**Experiment 3**					
Story	3.99	0.005	0.15	41.31	Story 4
Vowel	0.87	0.355	0.01	0.31 (3.14)	
Story × Vowel	0.59	0.67	0.02	0.20 (5.01)	
**Experiment 4**					
Story	5.65	<0.001	0.20	48.31	Story 5
Vowel	5.01	0.028	0.05	2.13 (0.47)	
Story × Vowel	1.99	0.101	0.08	0.79 (1.22)	


*Values in brackets in Bayes Factor column reflect evidence in favor of the null hypothesis – BF_*01*_. The Decrement column represents the point at which performance differs significantly from Story 1.*

The outcomes of the study show that there were two influences on target detection, the frequency with which specific vowel combinations appear in written language, and the story in which the targets occurred. The progressive decrement in performance across stories with the *ea* and *ea* combinations is consistent with the notion of resource depletion.

If depletion of resources was occurring over time (i.e., across stories), the low performance on the rarer vowel combinations could also be influenced by both natural language frequency and depletion of resources. To test this notion, we collected data from another 20 participants for whom the first and last stories were reversed, such that story 5 became story 1 and vice versa. Mean detection of the 11 rare combinations was 0.62 (*SD* = 0.15) when they appeared in the first story, and 0.47 (*SD* = 0.21) when they appeared in the final story, a difference that was statistically significant, *t*(10) = 3.89, *p* = 0.003, *d* = 0.74. Again, the decrement in performance between the first and last stories is consistent with the notion of resource depletion.

## Experiment 3

The outcomes of Experiment 2 show that when embedded in coherent text, detection of target items on the letter-e task is influenced by frequency of occurrence of the targets in the written language and the length of time participants have been engaged in the task. In the next experiment, we present the same target items in their original positions, but the words around them are randomized to eliminate the coherence of the narrative. Although the words are randomized, the stories have the same paragraph and sentence structure as in Experiment 2. If decrements in performance are driven in part by the language processes that underpin coherent text, then making the text less coherent may impact upon performance.

### Method

#### Participants

The total sample size was *N* = 28, with participants ranging in age from 18 to 63 years old, and having an average of 36.46 years (*SD* = 15.04 years). Females accounted for 71% of participants, and 64% were University students or graduates. While all participation for the experiment was voluntary, some (*n* = 12) were recruited from among 3-year psychology students, who subsequently received course credit for participating in the experiment. All other participants were recruited from the researcher’s (GTa) social and professional networks and received no incentive to participate in the experiment.

#### Materials

The materials were the same as those used in Experiment 2. The paragraph and sentence structure, as reflected in the use of commas and full stops (periods) remained the same in each story, as did the position of the target word. The words in each story were randomized such that the story was now incoherent, though individual words could be recognized as normal. An example of one paragraph is presented in **Figure 3**.

#### Procedure

The procedure was identical to that used in Experiment 2. That is, the five stories were presented as five slides on a PowerPoint presentation, where the participant verbally identified the target item by saying it aloud.

### Results and Discussion

The results of the experiment are summarized in **Figure 4**. As was the case in Experiment 2, recognition of the rarely occurring *oe, eo, ue,* and *eu*, combinations was relatively poor. For the frequent *ea* and *ee* combinations, performance again deteriorated across sessions, but in this instance, there was no difference in target detection between the two conditions. The outcomes of the 2 × 5 mixed factorial ANOVA, confirmed that performance did significantly deteriorate across the stories with strong evidence in favor of the experimental hypothesis. Performance was significantly below that observed on the first story by the fourth story. In contrast to Experiment 2, the difference in detection between *ea* and *ee* combinations, was not significant, with the Bayes analysis presenting strong evidence in favor of the null hypothesis. Likewise, there was strong evidence in favor of no interaction.

Reducing the level of coherence in the stimuli reduced the influence of pre-existing language features of the target items, in that there was now no difference in detection of frequently occurring *ea* and *ee* combinations. In addition, although performance deteriorated across sessions, it differed significantly from baseline only at Story 4.

## Experiment 4

Reducing the coherence of the text reduced the influence of pre-existing frequency effects. In the next experiment, we further reduce the coherence of the material by replacing the consonants in each story, with alternative random consonants, maintaining the vowels in each word, and the specific e-vowel combinations, sentence and paragraph structure. In so doing, we maintain the surface structure of the materials in Experiments 2 and 3, but the target vowel combinations are now embedded in random letter strings as was the case in Experiment 1.

### Method

#### Participants

Forty-nine volunteer participants were recruited from the social networks of five students who were enrolled in a research skills course. Each student was to recruit 10 participants but one recruited only 9 participants. The ages of the participants ranged from 18 to 66, with a mean of 32.43 years (*SD* = 12.44). Females made up 65% of the sample, and 61% had not attended university.

#### Materials

The materials were the same as those used in Experiment 2. The paragraph and sentence structure, as reflected in the use of commas and full stops (periods) remained the same in each story, as did the position of all vowels and specifically the target vowel combinations. The consonants in each story were replaced by random sets of consonants. Each story was now totally incoherent. An example of one paragraph is presented in **Figure 3**.

#### Procedure

The procedure was identical to that used in Experiments 2 and 3.

### Results and Discussion

The results of the experiment are summarized in **Figure 4**. Again, the infrequent e-vowel combinations were not well recognized, as was the case in the previous experiments. The mixed factorial ANOVA involving the frequent *ea* and *ee* combinations indicated that there was a significant decrement in performance across stories, but in this instance, performance was significantly worse from Story 1 only at Story 5. The ANOVA indicated that, in contrast to earlier experiments, target detection was significantly better for *ee* combinations than *ea* combinations, although the Bayes analysis suggested that the evidence in favor of this difference was not strong. Likewise, for the interaction between story and vowel combination, there was not strong evidence for either the null hypothesis or the alternative hypotheses.

## General Discussion

The current research was motivated by the call to validate intervention tasks in the sequential task paradigm as a way of resolving both conceptual and replication crises that currently plague self-regulation research. Of the intervention tasks available, we have focused on the letter-e task because it is both widely used and is amenable to measurement. In prior research, Arber et al. (2017) have shown that target detection does deteriorate on this task across time and has follow-on effects on working memory. In the current paper, we have examined some of the debated assertions that have been made concerning what are the causal factors in creating resource depletion. Experiment 1 was aimed at testing key assertions made by Baumeister and Vohs (2016) that depletion will only occur if there is a habit-forming component of the intervention tasks, and it is inhibition of that habitual response that is the causal mechanism for resource depletion. We considered other possible mechanisms that might be the causal factor, such as having to apply multiple rules, or the need to inhibit an automatic reading response. Thus, Experiment 1 was designed to (1) limit the influence of reading processes by presenting lines of randomized letters; (2) to assess the need for a habit-forming first step; (3) to assess the need for inhibition of an habitual response; and (4) to test the proposition that simply following multiple rules was the causal mechanism for the previously observed decrement in target detection across time.

The outcomes of Experiment 1 were unexpected in that there was no strong evidence for depletion across time where it was expected. While there were some weak indications of deterioration in the inhibition condition, the magnitude of the decline was very small relative to prior studies. The results of the current experiment do not provide compelling support Baumeister and Vohs (2016) assertion that habit-forming and inhibition are necessary to induce resource depletion. However, it is possible that the changes made to the presentation format induce fundamental changes to the task such that it becomes a simple visual scanning task rather than one that utilizes self-regulation resources. In sum, the results of Experiment 1 did not provide unequivocal information regarding the factors that produce substantial decrements in target identification across time that are associated with carry-over effects on other outcome tasks (Arber et al., 2017).

Experiments 2, 3, and 4 were designed to explore factors that might be responsible for producing large magnitude effects. The design systematically moved from coherent text that had shown performance decrements in the past to targets embedded in randomized consonants that did not result in performance decrements in Experiment 1. This manipulation was adopted to test the possibility that the decrement in performance was related to two factors, the frequency of specific e-vowel combinations in the language, and the effect of reading of coherent text. These changes necessitated the examination of performance on specific words.

The first outcome of these three experiments was that the pre-existing bigram frequency of the specific e-vowel combinations had an impact upon target detection in this task, just as it has in other word identification tasks. Rarely occurring combinations (*ae, oe, eo, ue,* and *eu*) were not as well identified as the more frequently occurring combinations (*ea* and *ee*). Moreover, the advantage of *ea* over *ee* combinations that were observed in coherent text, were eliminated when the words in the text were randomized, and were reversed when all the consonants in the story were randomized. This confirms the common-sense notion that language processes are important in this task. More importantly, it identified a confound in the stimuli used in the Arber et al. (2017) studies, in that the rare bigram vowel combinations were more prevalent in the latter stories. What Arber et al. (2017) interpreted as resource depletion effects were in part due to pre-existing bigram frequency effects. These findings indicate the need in future research for careful constructions of text stimuli to avoid such confounds and to present the stories in random orders across participants.

The second outcome of the experiments was that for the common *ea* and *ee* vowel combinations, target detection did deteriorate across stories in the latter three experiments, consistent with the notion of depleted resources. However, the coherence of the stimuli did appear to affect performance in that bigram frequency effects were attenuated as coherence was lost, but paradoxically, target detection appeared to improve as coherence was lost. An ANOVA that collapsed *ea* and *ee* target identification across the three experiments confirmed that there was a significant difference in performance, *F*(2,200) = 4.38, *p* = 0.014, $\eta_{p}^{2}$ = 0.04, with performance in the random consonants condition in Experiment 4 resulting in the highest level of performance, and the absence of a performance decrement until Story 5. This result, combined with the outcomes of Experiment 1, where there was no deterioration in performance, suggests a strong link between coherence and target identification indicative of inhibition of automatic reading responses as the mechanism driving the performance deficit. This explanation is broadly consistent with the assumptions of the strength model to the extent that repeated acts of inhibition deplete self-regulatory resources. However, the notion of inhibition of reading processes is yet to be posited as the causal mechanism underpinning resource depletion.

We would offer several caveats regarding the outcomes of the current studies. We have shown that in coherent text, target detection deteriorates when participants are required to follow multiple rules that involve inhibitory responses. We have not addressed the issue of whether or not simply searching for the letter e across stories in coherent text also produces decrements in performance. The critical assumptions of the strength model rely on differential patterns of deterioration between single and multiple rule conditions. Further research is needed to confirm or disconfirm this central assumption.

While we have linked reading and language processes to performance decrements in the letter-e task we have not directly demonstrated (or observed) that any resources have been depleted. The observed decrement in performance can also be explained by changes in motivation levels across stories (Inzlicht and Schmeichel, 2012), difficulties in goal maintenance (Dang et al., 2013), or a wide array of other alternative explanations. Even if resources have been depleted, we have not demonstrated that the depleted resources are those involved in self-regulation. Nor have we demonstrated that target identification is the appropriate measure of depletion of self-regulatory resources. Individual differences in overall levels of accuracy, the rate at which performance declines across stories, or the commission of self-regulatory errors such as responding to *ei* or *ie* combinations, are all alternative measures that can be derived from the task, and may be more predictive of self-regulatory depletion than target identification.

Taken together, the data presented across the studies do support the viability of the letter-e task; however, due to the limitations mentioned, alternative explanations need to be addressed in future research before any conclusions can be definitive. For now, we would suggest that if participants are searching for e-vowel combinations in coherent text and have to suppress a response to one of those combinations, then performance should decrease with continued engagement on the task. We have also shown that under conditions where the text is not coherent, there is comparatively little change in performance over time. This suggests that the need to inhibit automatic reading processes is a key driver of performance in this task as it is in other cognitive tasks like the Stroop task.

## Author Contributions

All authors were involved in the conceptualization and design of at least one experiment. LM, GTa, and SD: data acquisition. LM and GTe: analysis and initial drafting. LM, SD, GTa, JM, GTe, and MI: interpretation and revising. Final approval has been given by all authors and all accepted accountability for the work.

## Conflict of Interest Statement

The authors declare that the research was conducted in the absence of any commercial or financial relationships that could be construed as a potential conflict of interest.
